# From coral reefs to Joshua trees: What ecological interactions teach us about the adaptive capacity of biodiversity in the Anthropocene

**DOI:** 10.1098/rstb.2021.0389

**Published:** 2022-08-15

**Authors:** Katherine M. Lagerstrom, Summer Vance, Brendan H. Cornwell, Megan Ruffley, Tatiana Bellagio, Moi Exposito-Alonso, Stephen R. Palumbi, Elizabeth A. Hadly

**Affiliations:** ^1^ Department of Biology, Stanford University, Stanford, CA 94305, USA; ^2^ Stanford Woods Institute for the Environment, Stanford University, Stanford, CA 94305, USA; ^3^ Center for Innovation in Global Health, Stanford University, Stanford, CA 94305, USA; ^4^ Hopkins Marine Station of Stanford University, Pacific Grove, CA 93950, USA; ^5^ Department of Plant Biology, Carnegie Institution for Science, Stanford, CA 94305, USA; ^6^ Department of Global Ecology, Carnegie Institution for Science, Stanford, CA 94305, USA

**Keywords:** adaptive capacity, biodiversity, ecological interactions, Anthropocene

## Abstract

The pervasive loss of biodiversity in the Anthropocene necessitates rapid assessments of ecosystems to understand how they will respond to anthropogenic environmental change. Many studies have sought to describe the adaptive capacity (AC) of individual species, a measure that encompasses a species’ ability to respond and adapt to change. Only those adaptive mechanisms that can be used over the next few decades (e.g. via novel interactions, behavioural changes, hybridization, migration, etc.) are relevant to the timescale set by the rapid changes of the Anthropocene. The impacts of species loss cascade through ecosystems, yet few studies integrate the capacity of ecological networks to adapt to change with the ACs of its species. Here, we discuss three ecosystems and how their ecological networks impact the AC of species and vice versa. A more holistic perspective that considers the AC of species with respect to their ecological interactions and functions will provide more predictive power and a deeper understanding of what factors are most important to a species’ survival. We contend that the AC of a species, combined with its role in ecosystem function and stability, must guide decisions in assigning ‘risk’ and triaging biodiversity loss in the Anthropocene.

This article is part of the theme issue ‘Ecological complexity and the biosphere: the next 30 years’.

## 1. Introduction

The Anthropocene is defined by severe and far-reaching impacts of humans on the environment that are, in large part, the result of exponential population growth coupled with an increasing ecological footprint per capita. Factors novel to the Anthropocene that put stress on the well-being of biodiversity globally are myriad and include; habitat destruction, overharvesting, introductions of invasive species and novel pathogens, and global environmental pollution, majorly stemming from industrialization [1]. These extreme and rapid environmental impacts have led to a global biodiversity crisis dubbed the sixth mass extinction [2]. Today, species loss is happening at a rate estimated to be hundreds to thousands of times higher than in the last tens of millions of years [3]. This loss has been felt significantly among Earth's megafauna, as global defaunation trends show selection against the largest-bodied species [4], primarily as a result of direct harvesting for human consumption, but also by destruction of the habitats that support them [5]. This ‘trophic downgrading’ has a cascading effect resulting in changes to function and resilience of ecosystems globally [6].

**Figure 1.**
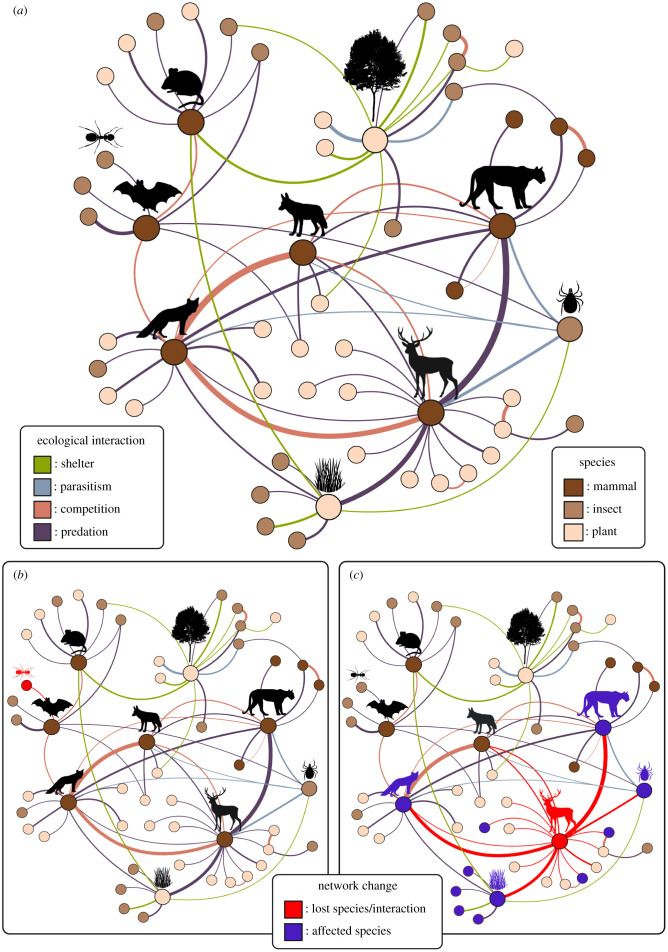
Ecological interactions network. (*a*) Illustrative intact network showing different ecological interactions occurring between species in the network coloured by the type of interaction. The thickness of the lines connecting the species in the network signify the strength of that interaction. (*b*) Example outcome of the impacts felt in the network by removal of a less connected and redundant insect species. The loss of the ant can be compensated for by the other insects connected to the bat. (*c*) Example outcome of the cascade of impacts resulting from the loss of a highly connected species with a unique role in the ecological network. The loss of the deer and therefore its interactions affect many other species and thus the overall stability of the network.

Apart from a few extreme, remote environments (e.g. polar deep sea, high elevation mountaintops or deep Earth fractures), species almost never exist singularly in an ecosystem; most communities are speciose. Intricate ecological networks composed of interactions such as those between primary producers and consumers, plants and pollinators, and predators and prey, evolved over long periods of time. Many ecological interactions are hierarchical, highly complex, and insufficiently understood, yet they ultimately drive survival of all species in a network. The extinction of a species is thus accompanied by the loss of its evolved complex interactions and may cause other species to go extinct (i.e. secondary extinctions). The loss of such ecological interactions may even precede the loss of a species, as drastic population reductions or hindrances to a species’ ability to fulfill their ecological role may be enough to have this effect. Consequently, the loss of interactions often occurs at a faster rate than species extinctions [7]. When these relationships collapse, significant knock-on effects impact the overall function and capacity of that ecosystem to support its inhabitants. Lost interactions must be compensated for in order to maintain the flow of energy, or the system must adjust by declining its trophic or community complexity and increasing metabolic efficiency. Relative to their evolution, or assembly time, the breakdown of these relationships, or disassembly, may occur abruptly with the loss of a key player in a densely linked ecological network. For example, ecological networks are far less resilient to (or more severely perturbed by) the extinction of generalized species than the extinction of specialist species [8]. Thus, secondary extinctions occur at a higher rate following the loss of highly connected, generalist species than less connected ones [9]. Disassembly of an entire system can therefore be caused by removal of a single highly connected species or one of critical functional importance, resulting in a ‘domino effect’ of downstream impacts (figure 1). The likelihood that these species and their interactions re-evolve fast enough to prevent ecosystem collapse is nil on the short timescales imposed by rapid changes in the Anthropocene. Earth's biodiversity must resort to other measures to buffer the effects of species loss on ecological networks and expand their capacity to respond to change.

Adaptive capacity (AC) has been formally defined as a species' ability to cope with or adjust to environmental change [10] via various mechanisms that can be grouped in four major categories (simplified from seven described in a ‘wheel’ of AC [11]): distribution and physical movement, plasticity and epigenetics, long-term genetic adaptation and ecological interactions. Each category encompasses numerous adaptive mechanisms that vary depending on the species being assessed. For example, plasticity and epigenetics could encompass behavioural or social structure and acclimation, while long-term genetic evolution represents adaptation and gene flow. Ecological interactions include symbioses, predator-prey relationships, setting population size and coevolution. These mechanisms operate at the level of species. However, adaptability and resilience of networks is achieved not only by the AC of species within the ecosystem but also by redundancy in ecological roles, such that the AC of an ecosystem is derived from its capacity to maintain functioning networks as well as the individual ACs of the species involved. Given the interconnectedness of Earth's ecosystems, assessments of AC must consider species' roles in their networks as well as the myriad interactions those species depend on for survival.

When we consider the next 30 years, adaptation by genetic evolution becomes infeasible for most species, apart from those with the shortest generation times, such as microbes. Therefore, the AC mechanisms relevant to the next several decades operate above the level of genome evolution, such as plastic responses via changes inbehaviour, phenotype and ecological interactions. AC in this sense is better described as a species' ability to respond to change in the next 30 years; whether that response is adaptive will take more time to manifest (i.e. must be heritable across generations, which are often longer than 30 years for many species, and be beneficial to future generations). Here we describe three distinct ecosystems to illustrate the manifold influences of ecological interactions on the ACs of species and consider the successfulness of certain human interventions aimed at bolstering those species' AC through this lens.

## 2. A sea of corpses or flourishing with life? Effects of warming on the reefs of Palau

The islands of Palau were formed from a combination of prehistoric volcanic events as well as (relatively) recent uplift of coral reefs in the South, creating a system of hundreds of islands that follow the submarine Palau trench to the East [12,13]. Two shallow lagoons extend from the main islands to the North and South and contain thousands of small patch reefs. Forereefs that fringe the archipelago in combination with many small patch reefs that dot the lagoons, create a wide variety of environmental conditions that vary in terms of depth, size, dissolved nutrients (due to varying proximity to land/areas of upwelling), temperature and pH. As is the case for many islands across the Pacific, the tropical reef ecosystems in Palau sustain a highly diverse set of flora and fauna, which in turn sustains human populations on the islands.

The combination of forereef habitats, as well as the inner patch reefs, creates a mosaic of abiotic and biotic conditions that corals must endure. However, survival is predicated both on the ability of the corals to withstand a wide range of conditions and on the ability of their microbial symbionts to do the same. The outcome of the interaction between the coral host and its symbiont must be favourable for both partners, which can be environmentally dependent. The collapse of this partnership leads to bleaching and ultimately the degradation of the habitat and its biological diversity and productivity. In order to make predictions about the AC of this system in the next 30 years, it is important to focus not only on the coral host, but also on the symbiont and their interactions across environments. For example, heritable genetic variation of the reef-building coral *Acropora millepora* accounts for some (10 to 15%) of the variance in coral bleaching datasets [14] but does not come close to predicting when a colony will bleach. This implies that considering the symbiont and host-symbiont interactions may increase the predictability of whether a coral colony will bleach during stressful conditions.

The changing climate of the Anthropocene has already led to selection and phenotypic changes in coral populations; however, it is unclear if these populations will be able to continue adapting to changing conditions at their current pace. While strong selective pressures can lead to rapid adaptive changes in as little as a single generation [15], coral generation times are on the order of years to decades [16]. Although fast-paced selection over a single generation will take many years to be reflected in future offspring, adaptations already segregating populations of the common tabletop coral (*Acropora hyacinthus*) are predicted to spread with modest rates of gene flow between populations [17]. Modelling studies suggest this result is broadly applicable across reef-building corals [18]. Further complications arise from the fact that heat stress acting on these populations is not uniform across the geographical ranges of these species. Some populations will inevitably not experience a warming event and its ensuing selective pressures but will produce larvae that can migrate to warmer regions, potentially swamping locally adapted alleles whose frequency increased due to a local warming event.

A significant source of adaptive variation in coral resides in the photosymbionts in the family Symbiodiniaceae. Although they are endosymbiotic, many coral species, including *Acropora hyacinthus*, transmit them horizontally, reacquiring partners each generation, typically near the time when larvae are competent to settle. Though the same evolutionary forces that shape host populations are at work in their symbionts, microorganisms can adapt to local conditions across smaller temporal and spatial scales than their hosts. Genetic evidence suggests that symbiont populations can match their local environment, which can change over a few meters in temperate systems with large environmental gradients such as the intertidal zone [19]. Additionally, Symbiodiniaceae generation time is short, allowing them to match temporal variation at timescales of weeks to months that would be impossible for their hosts, who typically live for years if not decades or centuries.

Ecological interactions that lead to ecosystem (in)stability could also arise from host-symbiont interactions. Perhaps not surprisingly, coral species that transmit symbionts horizontally interact with more symbionts than their vertically transmitting counterparts [20]. This includes many fast-growing, competitive species, especially those in the genus *Acropora*. If symbiont populations can adapt to warmer temperatures, it is possible that they can confer those advantages to the many host species with which they interact. Research on artificial selection to increase the pace of adaptation is underway [21]. Population genetic patterns suggest that symbiont populations do adapt to their host, even when horizontally transmitted, and increasingly stressful conditions could explain more specialized partnerships [19]. Thus, while multiple host species might associate with a common symbiont partner, coevolution to match either the internal host environment or a more stressful abiotic environment could reduce some of the benefits that hosts enjoy from a heat-adapted symbiont partner.

Host–symbiont interactions could alleviate some of the stresses of anthropogenic climate change on vulnerable populations, but management strategies that depend on these relationships will require care in ensuring that the nature of the interaction remains beneficial for both partners. How much those interactions expand or collapse the potential envelope of environmental conditions that the holobiont can tolerate is unknown. Studies of terrestrial plant-fungal associations suggest that they can be positive [22], but no comparable study exists in corals. There are emerging lines of evidence to suggest that the adaptive landscapes for both partners constrain the degree to which this relationship can maintain mutually positive outcomes as conditions warm. For example, if bleaching resistance emerges due to a parasitic symbiont that provides fewer benefits to the host, as has been proposed for corals associated with *Dursidinium* sp., instead of *Cladocopium* sp. after bleaching [23–25], the net benefit of the symbiosis to each partner could decline. Furthermore, if bleaching resistance comes at the expense of another trait such as growth [19], individuals might escape the acute risk to their fitness due to bleaching today, only to experience lower fitness later in life in the form of reduced reproductive output. Evolved responses to increased heat stress might not doom corals as a species or population but could profoundly restructure ecosystems where both productivity and persistence rely on the current balance of nutritional exchange between corals and their dinoflagellate partners. This balance could shift toward lower productivity or instability as conditions warm. The discrepancy between the rates of genetic evolution attainable by corals compared to their microbial symbionts sets up the potential for a symbiotic relationship to deteriorate into a parasitic one, or to breakdown completely. Thus, the AC of corals and the ecological network in which they are imbedded rely on the successful maintenance of mutually beneficial symbiotic interactions.

## 3. Joshua trees: a story of resilience (for now) despite few friends remaining

The charismatic Joshua tree (*Yucca brevifolia*) was once called the most repulsive tree in the vegetable kingdom by ninteenth century American explorers. They can be distinguished by their clumsy, thick ramifications ending in rosettes of short, sword-like leaves. According to the late Pleistocene fossil record, Joshua trees existed throughout the Sonoran and Colorado deserts, extending into northern Mexico [26]. While primarily hot and dry, the habitat persisted along a climatic gradient with higher temperatures in the South and at lower elevations, and lower temperatures at higher elevations in the North. The ecosystem was a biologically diverse landscape of creatures coexisting with the grasses, shrubs and succulents in a network of ecological interactions. The Joshua trees were reliably dispersed by the Shasta ground sloth (*Nothrotheriops shastensis*), which partially digested the fruits and dispersed the seeds in their nutrient-rich dung piles [27]. These bear-sized mammals were among the few megafauna in the desert habitat that could reach the fruits at the top of the unusually tall desert succulents known to grow up to 15 m. As the warming period of the Holocene began, Joshua trees faced many selective pressures. After the extinction of the ground sloth, majorly caused by big-game hunters [28], the distribution of Joshua trees retracted northward and moved to higher elevations with lower temperatures. The remaining range in the Mojave Desert is over 80% smaller than their historical habitat [26].

Despite drastic reductions in their range, Joshua trees remain a major hub for ecological interactions; many species rely on them for food and shelter. The leaves are short, narrow and sturdy, making them ideal nest-building material for birds and small mammals. Their nitrogen-rich young leaves are one of the few remaining food sources in dry seasons for herbivores like small rodents (genera *Neotoma* and *Peromyscus*) [29]. Their fruits are often used by insects for food and to lay their eggs, and birds feast on these insects and eggs [30]. Though Joshua trees provide many ecological services, they rely on other organisms for sexual reproduction and dispersal. In the roughest conditions of the Mojave Desert, a unique relationship between the Joshua tree and its exclusive pollinator, the Yucca moth (*Tegeticula synthetica*) [31], evolved to a mutualistic dependency. Female moths lay eggs inside the ovary of the flower, ensuring the future of their larva with a nutrient-rich environment, while deliberately depositing pollen on the stigma [31,32]. While the larvae will consume some of the seeds, Joshua trees depend on the remaining ones to maintain gene flow as a source of genetic diversity. Today, packrats and rodents fill the role left by the ground sloth, acting as the primary dispersers of Joshua tree seeds. Most fruits are accessed in the canopy by white-tailed antelope squirrels (*Ammospermophilus leucurus*) and fallen seeds are taken by rodents like the Merriam's kangaroo rats (*Dipodomys merriami*). In abundant seasons, the scatter-hoarding rodents bury the seeds in their caches, protecting them from desiccation and providing a suitable site for germination [33]. In this case, the AC of the ecosystem resulted in another species filling the role of seed disperser for Joshua trees, but even similar ecological roles can have inequivalent downstream effects. Indeed, Joshua trees face a drastic reduction in dispersal capability with their new rodent dispersers compared to the ground sloth, thus dramatically slowing their migration to suitable habitats [34].

As temperatures continue to rise, the area of suitable habitat will lessen. Increased drought occurrence, along with the invasion of combustion-prone bushes, have drastically increased the number of fires in the area [35,36]. Joshua trees, like many desert species, lack fire response adaptation and have an 80 to 90% mortality rate when burned [37]. Young trees are the most susceptible since their meristem is nearest to the soil [38]. Over the past four decades, there has been a 250.61% increase in acreages burned by wildfires in Joshua tree habitats [30], increasing the mortality rate in exposed populations and further limiting the species' ability to disperse and experience population growth. The popularity of Joshua Trees, both the National Park and the city, has brought a large increase in the human population and with it a rise in land-use demand. Ironically, this has also led to habitat destruction of the Mojave Desert and ultimately, Joshua trees. Joshua trees have locally adapted to various environments across their native range by responding to different selective pressures [39]. Some of these locally adaptive strategies have fitness trade-offs in different environments. Therefore, the best solution to help Joshua trees adapt to new habitats is to identify both the genetic variants that contribute to the adaptive strategies and the habitats they match, and then to strategically migrate those genotypes with the best prognosis for a given new habitat (i.e. an informed genetic rescue). There are ongoing efforts to identify genotypes among individuals of present populations that have adapted to varying desert climates through common garden experiments, but they are still in their nascent stages.

Multiple models of future distribution predict a substantial decline in the suitable climates remaining for the Joshua tree [26,40,41], mainly due to severe increases in temperature predicted in the Southwest of North America. Some predictions expect 90% of extant populations to go extinct in the coming decades [41]. Either temperatures need to remain in a range that Joshua trees can tolerate, which can only happen if humans limit carbon emissions drastically, or Joshua trees must migrate to more suitable habitats. Given their limited dispersal capabilities and long generation time, it is unlikely they can migrate quickly enough to outpace the projected rise in temperature on their own. Even if they move successfully, it is unclear whether their obligate pollinator, the Yucca moth, will be attracted to and survive in the new location. The outlook is not good here either, as studies already show that the mutualistic relationship is less successful at higher elevations, which is one way the trees are projected to move, as cooler climates often accompany higher elevations [42]. However, there still remains a chance that novel replacement interactions may arise in the new environment, which will likely expose the trees to new species and interaction networks. Joshua trees are another example of how the AC of a species is a rather abstract concept until studies consider its ecological interactions.

## 4. A historic park with a history of network perturbations: invasive species in Yellowstone

Yellowstone National Park, the first of its kind, was established in 1872, declaring the land too valuable in its natural wonders to be developed. The designation was made ‘for the benefit and enjoyment of the people’, but it ended up benefiting a far more expansive network of species [43]. The road to protecting the lands was not without its bumps, as expansive wildlife control efforts, including culling of wolves, elk, bison and other animals, whose population levels were deemed a ‘problem’, have occurred extensively over the years. Famously, wolves were eradicated from the park in 1926 but later reintroduced following the Endangered Species Act of 1973 in efforts to restore natural ecosystem interactions and functioning [44]. In 1963, the Leopold Report (named after prominent ecological scientist, Aldo Leopold) was released by a national park advisory group recommending that parks ‘maintain biotic associations' within their ecosystems [43]. Significant changes to the networks of the Greater Yellowstone Ecosystem (GYE) have occurred over the Park's history (many of which were the direct result of human actions), thus making it an ideal case study to discuss the impacts of invasive species and network rewiring on the AC of species and ecosystems.

In 1994, a breeding population of non-native lake trout (*Salvelinus naymaycush*) was discovered in Yellowstone Lake [45]. Over the next several decades, widespread shifts in ecosystem function across trophic levels occurred across the GYE. The native Yellowstone cutthroat trout (YCT; *Oncorhynchus clarki*) populations suffered, not only from niche competition but from direct predation by the lake trout [46]. Animals that had previously been able to consume the YCT occupying the upper layers of Yellowstone Lake were unable to prey on the deeper-inhabiting invasive lake trout and were forced to shift their diets. One study estimated that 14 to 21% of all grizzly bears (*Ursus arctos horribilis*) in the GYE relied on the YCT in Yellowstone Lake and its tributaries for food. In the years following the introduction of lake trout, fishing by bears had decreased in nearly every documented tributary [47]. The lack of fish caused bears to increase predation on elk calves (*Cervus canadensis*), which in turn affected the demography of the elk [48]. Another predator of YCT, the osprey (*Pandion haliaetus*), experienced drastic reductions in population size due to the invasion of lake trout. Osprey nest counts at Yellowstone Lake averaged 38 between 1987 and 1991, but there were only 3 nests present between 2013 and 2017 [46]. Additionally, from 2008 to 2011, not a single osprey was successfully fledged [46]. North American river otters (*Lontra canadensis*) in the tributaries of Yellowstone Lake have been shown to have markedly long and late breeding seasons, likely in conjunction with YCT spawning [49]. However, recent eDNA surveys of historic YCT spawning streams in Yellowstone Lake found no evidence of river otters [50].

The decline in YCT also affected non-animal aspects of the GYE. Because the lake trout did not travel upstream to breed, nutrient profiles of the streams serving the lake changed, most notably in soil nitrogen deposits fueled by dead YCT after their journey upstream [51]. Concurrently, ammonium uptake by phytoplankton in Yellowstone Lake increased, phytoplankton assemblages shifted to favour larger-bodied species, and overall biomass of phytoplankton significantly decreased. The longnose sucker (*Catostomus catostomus*), which consumes phytoplankton, thus experienced a marked decrease in population. Decreased phytoplankton biomass also resulted in increased water clarity, which in turn caused water temperature to rise [46]. This lucidly illustrates myriad downstream effects caused by the removal of a species with an important role in a highly connected ecological network—a role that an invasive replacement species could not fill.

Niche conservatism defines the degree to which plants and animals retain their function or position and related ecological traits through space and time within an ecological community. Niche conservatism above the species level remains one of the most robust ways to ascertain the community function of species [50,52]. Primary factors controlling niche conservatism are intrinsic and inherited life-history traits (e.g. body size, diet, reproductive rate, etc.), whereas niche conservatism at the species level may reflect underlying environmental controls and competition. Thus, in communities with speciose genera, conservation of ecosystem function may best be accomplished by substitution of species within genera. Not all species within a genus are equal substitutes, however, as demonstrated by the story of invasive lake trout in Yellowstone, wherein even two very closely related species did not perform adequately redundant ecosystem services. Nevertheless, substitution of sister species with similar life histories may indeed provide a parsimonious way to predict future scenarios during extinction events [52].

## 5. Conclusion

It has been said that species have three ways to respond to the extreme environmental change resulting from anthropogenic actions in the Anthropocene: move, adapt, or die; ‘the ‘MAD’ response’ [53]. We have discussed the limitations to genetic adaptation on such short timescales, and how physical movement is complicated by pertinent ecological interactions that must shift in parallel for migrations to be adaptive solutions. When individuals or populations move, they will often encounter novel assemblages of species and must integrate symbiotically with the new network to maintain ecosystem functioning. We have shown that when species fail to integrate or to fill an open niche effectively, they can throw an entire ecosystem off balance by disrupting important relationships and preventing other key players from fulfilling their essential functions, resulting in the negative connotation surrounding the term ‘invasive species’. Those species that cannot move will be forced to adapt quickly, potentially by forming new beneficial relationships or via other plastic changes that occur above the level of the genome. The breadth of plastic responses and how they occur are not yet well-understood, partially because they encompass a wide range of AC mechanisms that operate at different timescales and are only adaptive if they confer a fitness advantage, which is context-dependent (i.e. what is adaptive today may not be following future change). However, examples of plastic responses can be seen in changes in the timing of developmental stages, reallocation of resources and dietary flexibility, among others; epigenetic variation may be an underlying factor in the expression of such plastic phenotypes [54].

The environments, ecosystems, and interaction networks of the future will be defined by the rapid changes and far-reaching impacts of the Anthropocene and will likely incorporate many novel features. Thus, we advocate for the expansion of the meaning behind current ‘re-’ lexica; re-storing, re-wilding, re-networking, re-establishing, which all imply re-creating a past situation. These terms better serve us when they acknowledge the necessity of responding to change, potentially through the creation of novel networks and AC strategies. This shift in thinking, along with more thorough understanding of the many dimensions of AC, will aid us in establishing the most effective conservation plans. A more holistic perspective of AC is one that combines the consideration of ecological interactions and functions with species-level factors contributing to survival. This perspective will better guide us in assessing the risk of species extinction and ensuing ecosystem collapse, thereby enhancing our ability to curtail biodiversity loss in the Anthropocene.

## Data Availability

This article has no additional data.
